# Utilization of renewable biodiesel blends with different proportions for the improvements of performance and emission characteristics of a diesel engine

**DOI:** 10.1016/j.heliyon.2023.e19196

**Published:** 2023-09-04

**Authors:** Yanhui Chen, Jian Zhang, Zhiqing Zhang, Weihuang Zhong, Ziheng Zhao, Jingyi Hu

**Affiliations:** a Mechanical and Engineering Department, Guangxi Vocational College of Water Resources and Electric Power, Nanning, 530023, China; b Guangxi Earthmoving Machinery Collaborative Innovation Center, Guangxi University of Science and Technology, Liuzhou, 545006, China; c Guangxi Key Laboratory of Automobile Components and Vehicle Technology, Guangxi University of Science and Technology, Liuzhou, 545006, China

**Keywords:** Diesel engine, Biodiesel blends, CONVERGE, Performance and emission characteristics, Combustion

## Abstract

This work investigated and compared the impact on performance and emission characteristics of diesel engine fueled with five different proportions of biodiesel blends. Firstly, the three-dimensional simulation software CONVERGE was used to create a 3D simulation model of in-cylinder combustion for a diesel engine. Secondly, the experimental data of cylinder pressure and NO_x_ emissions at 50% and 100% loads were employed to verify the simulation model. Finally, the combustion processes of blends with proportions of 0%, 5%, 10%, 15%, and 20% biodiesel were simulated and compared by using the model. The study showed that the brake thermal efficiencies (BTEs) of biodiesel blends with 5%, 10%, 15%, and 20% of biodiesel were increased by 1.24%, 1.89%, 3.13%, and 3.82% at 50% load, respectively, compared with pure diesel. In addition, the soot emissions were decreased by 1.20%, 2.64%, 3.88%, and 4.65%, respectively. However, as the proportion of biodiesel in the biodiesel blends increased, the brake specific fuel consumption (BSFC) and NO_x_ emissions increased. At 50% load, the BSFCs of biodiesel blends with 5%, 10%, 15%, and 20% of biodiesel increased by 0.61%, 1.34%, 1.42%, and 2.17%, respectively, compared with pure diesel. Additionally, the brake powers (BPs) were decreased by 0.64%, 1.31%, 1.88%, and 2.62% at 100% load, respectively.

## 1 Introduction

With the global energy crisis [1], climate change and pollution problems [2], the energy policy adjustment has become a new idea for energy development in some countries [3]. However, the pollution emissions from vehicle engines are an important contributor to environmental pollution [4,5]. The produced nitrogen oxides (NO_x_), carbon monoxide (CO), hydrocarbons (HC), and some other solid particles in the operation process are bad for human health [6,7]. It is necessary to decrease engine emissions by using some methods, such as EGR, alternative fuels, and so on [8,9]. In terms of alternative fuels, there is a commitment to finding a renewable resource with a lower carbon than fossil fuels [10]. The emergence of a green energy source, namely biofuels, is valued by many countries [11]. The biofuel industry has developed rapidly in recent decades [12]. The development was firstly in Brazil and the United States, and then China and the European Union also made great efforts to develop biofuels [13]. The European Union had stated in its development plan for biofuels that biofuel consumption had accounted for 10% of the total transportation fuel consumption in 2020. In November 2022, the United Nations Climate Change Conference at COP27 had be held in Egypt, where world leaders discussed about reducing global warming gas emissions to combat climate change [14]. The United Nations Secretary-General Antonio Guterres had reported that more works were needed to be done to significantly decrease emissions. The use of fossil fuels will lead to more pollution emissions [15,16]. Therefore, protecting the environment and reducing emissions from vehicles has become an urgent issue [17].

In general, biodiesel is a renewable and clean alternative fuel. The content and concentration of polycyclic aromatic hydrocarbons (PAHs) in biodiesel are lower than that in traditional fuels, which is a Carcinogen [18]. Thus, lots of researchers have carried out the research on biodiesel. For example, Zhang et al. [19] found that biodiesel's CO and soot emissions were decreased by 30% and 70% compared with diesel. In addition, Zhang et al. [20] demonstrated that utilizing a dual-fuel approach could significantly decrease combustion duration (CD) while also enhancing BTE. Goga et al. [21] investigated and analyzed a dual-fuel engine using a combination of diesel, rice bran biodiesel, n-butanol as liquid fuels, and biogas as gaseous fuels. The results revealed that compared with conventional diesel, the BTE for dual-fuel was decreased by 14.68% on average. Additionally, CO and HC emissions were increased by 45.58% and 17.74%, respectively. Compared with conventional diesel, the NO_x_ and smoke emissions were reduced by 39.06% and 46.85%, respectively. Senthilkumar et al. [22] investigated the relationship between cashew nutshell biodiesel (C100) and acetylene (Acetylene) and ignition characteristic of diesel engines. According to their findings, when the diesel engine fueled with acetylene gas with the base fuel, (C100) the soot, HC, and CO_2_ emissions were reduced by 6.87%, 9.7%, and 18.1%. However, the NO_x_ emissions were increased by 11.1%. Additionally, compared with pure biodiesel at all flow rates, the acetylene-biodiesel blend had a higher BTE. Furthermore, the incorporation of acetylene gas into the base fuel (C100) augmented the heat release and peak pressure. In similar studies, the impact of Chlorella biodiesel blends on emission and combustion characteristics was studied by Sathish et al. [23]. The experiments were conducted with a naturally aspirated dual-fuel diesel engine at 1500 rpm with different loads. The findings indicated that the blended fuel with 20% of Chlorella microalgae biodiesel had higher cylinder pressure, lower combustion time, and ignition delay compared with diesel. The emission and combustion characteristics had been improved.

The performance of TME-diesel blends with different volume proportions of biodiesel (e.g. 5, 10, 15, 20, and 25) were compared with pure TME (Thumba methyl ester) and pure diesel. The results showed that the TME-diesel blends with 20% biodiesel had the lower emission and higher BTE [24]. In addition, Jatropha curcas oil was selected as a feedstock for biodiesel and blended with diesel fuel at 20%, 40%, 60%, 80%, and 100% by volume (B20, B40, B60, B80, and B100) to evaluate the performance, emission characteristics of diesel engines. The results showed that the high proportion of biodiesel by volume led to lower BP and more drawbacks. The authors recommended the use of lower proportions of biodiesel up to 20% by volume as an alternative fuel [25]. Based on the above results, a suitable biodiesel blends can reduce the pollutant emissions and improve the engine combustion. The engine performance can be optimized while simultaneously minimizing harmful emissions by the method [26,27]. Therefore, replacing fossil diesel with a certain proportion of biodiesel has become a trend in the future development of diesel engines.

However, there are some problems before large-scale use. Uyumaz et al. [28] experimented with the diesel engine fueled with biodiesel-diesel blends with 10% and 20% poppy oil biodiesel (OP10 and OP20). The result showed that the BTEs of OP10 and OP20 were decreased by about 6% and 13%, respectively, compared with diesel at 75% engine load. This was due to the poppy oil biodiesel's lower caloric value. At full load, the NO_x_ emissions for OP10 and OP20 were 2.9% and 5.98% higher than pure diesel, respectively. At full load, compared with diesel fuel, OP10 and OP20 decreased CO emissions by 14% and 17.42%, respectively. The effect of blending corn oil methyl ester with pure diesel fuel on the combustion and emission of diesel engines was studied [29]. The results showed that the higher oxygen content in the blended fuel increased NO_x_ emissions, but decreased CO and HC emissions. And the BSFC of B10 biodiesel blends was increased by only 2%. While B20 and B30 were used, the BSFCs increased by 4% and 6%, respectively. Similarly, Srikanth et al. [30] examined the milk waste (MDWS) biodiesel as an alternative fuel for diesel engines. The findings indicated that the addition of biodiesel to fossil diesel led to an increase in BTE due to the enhanced combustion caused by the presence of oxygen in the biodiesel. However, an increase in BSFC was also found for the biodiesel blends at full load. More specially, NO_x_ emissions for the B50 were 32% higher than that for diesel. Additionally, Reksowardojo et al. [31] pointed out that the use of a high proportion of biodiesel could decrease CO, HC, and soot emissions. However, as the proportion of biodiesel increased, NO_x_ emissions increased [32]. In a similar conclusion, the results of the study by Yusuf et al. [33] indicated that the addition of biodiesel led to an increase in NO_x_. This leads to lung tissue damage and causes respiratory diseases [34,35].

As mentioned above, the biodiesel blend can improve the performance and emission characteristics. The blend proportion plays a crucial role in the improvements of performance and emission characteristics. Numerical simulation is extensively used in diesel engine research to overcome the drawbacks of lengthy bench test cycles and high costs. Based on this, this paper conducts the research by the numerical simulation. Firstly, this study established a 3D simulation model of diesel engines utilizing CONVERGE software. Then the model was validated by experimental results and employed to investigate the impacts of different blending proportions on diesel engine performance, combustion, and emission characteristics. The purpose of this study was to investigate the potential advantages of different proportions of biodiesel blends and improve engine efficiency and pollution emissions. Through the collection and analysis of experimental data, it would provide a strong scientific basis to further optimize the use of biodiesel blends and provide valuable information for researchers. In addition, some literatures are controversial on the impact of biodiesel proportion on parameters such as BTE, and the findings of this paper provide a reference for this. This study has academic implications and concrete practices for achieving source reduction of diesel vehicles.

## 2 Materials and methods

### 2.1 Numerical model of diesel engine

(1) Mass conservation equation

The mass conservation involved in fluid motion means that the inflow mass of the fluid is the same as the outflow mass. If the inflowing and outflowing fluid masses are not equal, the difference is the fluid mass accumulated in the flow space. Thus, the fluid mass conservation law can be expressed as the mass increase of fluid microelements per unit of time equals the net mass of simultaneously inflowing microelements. In fluid computational mechanics, the continuity equation is the performance of fluid mass conservation [36]. Equations are as follows:

(1) $$dM_{z}/d\theta =\sum (dM_{q}/d\theta )-\sum (dM_{i}/d\theta )-dM_{o}/d\theta +dM_{r}/dt$$

where *θ* is the angle of the crankshaft, ° CA; *M*_*z*_ is the total mass of substances in the cylinder, kg; *M*_*q*_ and *M*_*r*_ are gas and fuel masses entering the cylinder, kg; *M*_*i*_ and *M*_*o*_ are the mass of gas entering and leaving the cylinder, kg; *t* is the time, s.

(2) Energy conservation equation

The principle of energy conservation states that the sum of the heat entering the micro-element and the work done on the micro-element by body and surface forces is equal to the rate of energy increase within the micro-element. Equations are as follows [37]:

(2) $$d\left(M_{z}\cdot e\right)/d\theta =\sum (M_{q}/d\theta )\cdot h_{e}+dQ_{j}/d\theta -p_{i}\cdot \left(dV/d\theta \right)-\sum (dQ_{s}/d\theta )\;-\sum (dM_{i}/d\theta )\cdot h_{o}-\left(dM_{o}/d\theta \right)\cdot h_{u}-q_{o}\cdot f\cdot \left(dM_{r}/dt\right)$$

where *e* is the specific internal energy in the cylinder, J/kg; *h*_*e*_ is the specific enthalpy of the inflow gas, J/kg; *h_o_* is the specific enthalpy of the outflow gas, J/kg; *Q*_*j*_ is the heat release of fuel, J; *p*_i_ is the pressure in the cylinder, Pa; *V* is the working volume, cm^3^; *Q*_*s*_ is the heat loss, J; *h*_*u*_ is the specific enthalpy of the leaking gas, J/kg; *q*_*o*_ is the fuel latent heat of vaporization, J; *f* is the fraction by mass of vaporized fuel.

(3) Ideal gas equation of state

The equation of the state of ideal gas describes the relationship between density, pressure, and temperature. During 3D simulation, the ideal gas state equation is used to calculate the gas stat [38]. Equations are as follows [39]:

(3) $$p_{i}=(1/v)\cdot M_{z}\cdot R_{a}\cdot T_{q}$$

where *R*_*a*_ is the gas constant, J/(K·kg); *T*_*q*_ is the cylinder block temperature, K.

(4) Component transport equation

The sum of the net diffusive flow through the system cross-section and the production rate of the components generated through the chemical reaction is equal to the rate of change of the mass of the chemical components in the system concerning time, which is known as the law of conservation of components. The operation of the diesel engine is accompanied by gas exchange, fuel composition consumption, and combustion product generation. In the overall system, the component transport equation can be derived from the following expression [40]:

(4) $$\partial \rho_{m}u_{i}/\partial x_{i}+\partial \rho_{m}/t=S_{m}+\partial \left[\rho D\left(\partial Y_{i}/\partial x_{i}\right)\right]/\partial x_{i}$$

(5) $$Y_{m}=N_{m}/N_{tt}=\rho_{m}/\rho_{tt}$$

where *D* is the coefficient of mass diffusion, m^2^/s; *S*_*m*_ is the formation rate of component *m* per unit time; *ρ*_*m*_ is the fluid density, kg/m^3^; *u*_*i*_ is the velocity vector, m/s; *Y*_*i*_ is the fraction by mass of component *m*, %; *N*_*m*_ is the mass of the microelement control volume component *m*, kg; *N*_tt_ is the whole mass of the micro-element control body component, kg.

(5) Spray model

Fuel spray is impacted by parameters such as spray pattern, spray environment pressure, and orifice parameter. To determine the time rate of droplet size change in this project, the Frossling model is selected as the evaporation model. The KH-RT model is used as a fragmentation model and the dynamic droplet resistance model is used as a gas resistance model [41]. The KH-RT model can be derived from the following expression [42]:

(6) $$R_{r}=C_{x}\lambda$$

(7) $$\tau =3.7C_{y}r_{w}/\lambda \psi$$

(8) $$\lambda =9.02r_{w}\left(1+0.45O_{H}^{0.5}\right)\left(1+0.4T_{c}^{0.7}\right)/{\left(1+0.865We^{1.67}\right)}^{0.6}$$

(9) $$\psi =\left[(0.34+0.38We^{1.5})/\left(1+O_{a}\right)\left(1+0.4T_{c}^{0.6}\right)\right]\sqrt{\sigma /\rho_{w}r_{A}^{3}}$$

where *R*_r_ is the initial radius, m; *C*_*x*_ and *C*_y_ are constants of the injector; *ψ* is the index of the wave height; *λ* is the wavelength, m; *τ* is the existence time of the oil beam, s; *r*_w_ is the radius of the droplet, m; *O*_*a*_ is the Anseger number of liquid drops; *σ* is the tension of the surface; *T*_*c*_ is Taylor number; *ρ*_w_ is the liquid's density, kg/m^3^; *We* is the Weber number of the continuous phase.

(6) Turbulence model

This paper employs the RNG k-ε turbulence model to simulate the in-cylinder flow field. The turbulent kinetic energy *k* and turbulent dissipation rate *ε* can be obtained using the following expression [43]:

(10) $$\partial \rho k/\partial t+\partial \left(\rho u_{i}k\right)/\partial x_{i}=\tau_{ij}(\partial u_{i}/\partial x_{i})+\partial \left[\left(\mu_{e}/Pr_{k})(\partial k/\partial x_{j}\right)\right]/\partial x_{j}-\rho \varepsilon +S_{o}$$

(11) $$\partial \rho \varepsilon /\partial t+\partial \left(\rho u_{i}\varepsilon \right)/\partial x_{i}=\partial \left[\left(\mu e/\mathrm{Pr}\;\varepsilon \right)\left(\partial k/\partial x_{j}\right)\right]/\partial x_{j}-C_{\varepsilon m}\rho \varepsilon \left(\partial u_{i}/\partial x_{i}\right)+\left[C\varepsilon n\tau_{ij}(\partial u_{i}/\partial x_{j})-C_{a}\rho \varepsilon +\varepsilon CaSo\right]\left(\varepsilon /k\right)+S-\rho R$$

where *μ*_*e*_ is the effective viscosity of the fluid, Pa·s; *Pr*_*ε*_ *and* Pr_*k*_ are the Prandtl numbers in the *ε* equation and the *k* equation, respectively; *τ*_*ij*_ is Reynolds stress, Pa; *C*_*εm*_、*C*_*εn*_, and *C*_*a*_ are empirical constants; *S*_*o*_ is the source term.

(7) Combustion model

The SAGE model in CONVERGE software is chosen to simulate the combustion process of the fuel in this paper. The diesel-biodiesel mechanism used is that of Liu et al. [44]. This chemical reaction mechanism contains 106 reactants and 263 chemical reactions. Equations are as follows:

(12) $$\sum_{n=1}^{N}v_{n,r}^{\prime }x_{n}=\sum_{n=1}^{N}v_{n,r}^{\prime \prime }x_{n}\;\left(r=1,2,...R\right)$$

(13) $$\bar{\omega }=\sum_{r=1}^{R}v_{n,r}q_{r}\;\left(n=1,2,...N\right)$$

(14) $$v_{n,r}^{\prime \prime }-v_{n,r}^{\prime }=v_{n,r}$$

(15) $$q_{r}=k_{fr}\overset{N}{\underset{n=1}{\Pi }}{\left[X_{n}\right]}^{v_{n,r}^{\prime }}-k_{rr}\overset{N}{\underset{n=1}{\Pi }}{\left[X_{n}\right]}^{v_{n,r}^{\prime \prime }}$$

where *v′′*_*n,r*_ and *v′*_*n,r*_ are the products and reactants' stoichiometric coefficients of component *n* and reaction *r*, respectively; *N* is the total number of substances; *R* is the total number of reactions; *x*_*n*_ is the component *n*'s chemical symbol; [*X*_*n*_] is the molar concentration of substance *n*; *k*_*rr*_ and *k*_*fr*_ are the reaction rate coefficients of the reverse and forward reactions *r*.

(8) Heat transfer model

During the operation of the engines, there are always heat transfers taking place. In order to simulate this process more accurately in CONVERGE software, the OʹRourke and Amsden model was selected in this paper [45]. The heat exchange model can be derived from the following expression [46]:

(16) $$k(\text{d}T/dx_{i})=\left\{ \begin{array}{c} \mu c_{p}\left(T_{b}-T_{a}\right)n_{i}/\mathrm{yP}r_{t}\;\;y^{+}<11.05\\ \left[\rho c_{p}\mu_{\tau }T_{b}\;\ln\left(T_{b}/T_{a}\right)n_{i}\right]/\left[2.1\cdot \ln\left(y^{+}\right)+2.513\right]\;y^{+}>11.05 \end{array}\right.$$

where *k* is the coefficient of conductivity of the molecule; *T*_*b*_ is the temperature of the wall, K; Pr_*t*_ is the Prandtl number of the molecule; *T*_*a*_ is the temperature of the liquid, K; *μ*_*τ*_ is the shear speed; *y*^*+*^ is the dimensionless distance.

(9) Emission model

The extended Zeldovich NO_x_ model is chosen in this paper [47]:

(17) $$O_{2}↔2O$$

(18) $$N+O_{2}↔\text{NO}+O$$

(19) $$O+N_{2}↔\text{NO}+N$$

(20) OH+N↔H+NO

### 2.2 Experimental scheme and verification

#### 2.2.1 Main parameters and boundary conditions of diesel engine

The temperature boundary conditions are given empirically and the relevant settings are as follows, the temperatures of the piston, cylinder head, cylinder wall, and spray nozzle are 553 K, 523 K, 373 K, and 550 K, respectively. The velocity boundary condition is considered to be the piston motion velocity as the actual piston movement velocity at the calculated speed.

The turbulent kinetic energy and turbulent length scale in the combustion chamber can be derived from the following expression [48].

(21) TKE=(3/2)×u

(22) u=0.7×2×h×(n/60)

(23) $$TLS=h_{v}/2$$

where *u* is the turbulent pulsation velocity, mm/s; *n* is diesel engine speed, rpm; *h* is diesel engine stroke, mm; *h*_*v*_ is the maximum valve lift, mm.

The major parameters and boundary conditions of the engine are listed in Table 1.

**Table 1:** Engine parameter.

Parameter	Value	Parameter	Value
Cylinder bore × Stroke (mm)	190 × 210	Initial cylinder turbulent kinetic energy (m^2^/s^2^)	18.375
Connecting rod (mm)	410	Compression ratio	14
Rated speed (r/min)	1000	Initial pressure of air inlet (MPa)	0.193
Number of fuel injection holes	8	Effective power (kW)	220
Nozzle radius (mm)	0.28	Injection angle (°)	150

#### 2.2.2 Fuel characteristics

This study used rapeseed oil methyl ester (RME) as a biofuel blended with diesel fuel. In the paper, RME can be obtained by the transesterification method. The transesterification reaction was carried out at a molar ratio of oil to methanol of 1:6 and 1% wt of potassium hydroxide as a catalyst [49]. RME contains five main components, whose chemical formulas and volume fractions are shown in Table 2. To find out more about biodiesel blends, check out the previous work carried out by our team [50,51].

**Table 2:** Chemical formula and volume fraction of the five components of RME [49].

Components	Chemical formula	Volume fraction
Methyl oleate	C_19_H_36_O_2_	65.18
Methyl linoleate	C_19_H_34_O_2_	22.27
Methyl palmitate	C_17_H_34_O_2_	3.57
Methyl stearate	C_19_H_38_O_2_	0.87
Methyl linolenate	C_19_H_32_O_2_	8.11

In addition, diesel (D100) and four different proportions of biodiesel blends D95B5 (5% biodiesel + 95% diesel), D90B10 (10% biodiesel + 90% diesel), D85B15 (15% biodiesel + 85% diesel) and D80B20 (20% biodiesel + 80% diesel) were defined. In this study, 2,6-di-tert-butylphenol was blended with biodiesel blends at a concentration of 500 ppm. The blends were stirred at 1200 rpm for 30 min at room temperature to allow for thorough blending. The input physical properties of the model were processed using constant values. Table 3 lists the fuel characteristics [52,53].

**Table 3:** Fuel characteristics.

Fuel	Diesel	Biodiesel (RME)	D95B5	D90B10	D85B15	D80B20
Flash point temperature (°C)	67	168	72.05	77.1	82.15	87.2
Oxygen volume fraction (%)	0.0	10.7	0.535	1.07	1.605	2.14
Density (g/m^3^, at 20 °C)	0.82–0.86	0.882	0.8231–0.8611	0.8262–0.8622	0.8293–0.8633	0.8324–0.8644
Latent heat of vaporization (kJ · kg ^−1^)	253.31	273.36	254.31	255.315	256.32	257.32
Viscosity (MPa · s, at 20 °C)	3.0–8.0	4.556	3.08	3.16	3.23	3.31
Cetane number (CN)	45–66	53.88	45.444	45.888	46.332	46.776
Low heating value (MJ · kg^−1^)	42.5	39.53	42.3515	42.203	42.0545	41.906

#### 2.2.3 Grid independence verification

**Fig. 1:**
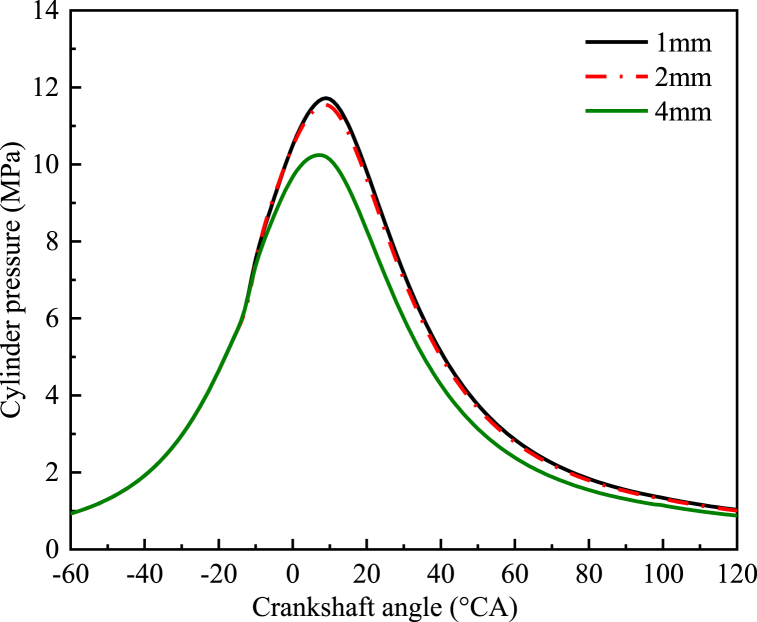
Cylinder pressure curves for three different grids.

According to the symmetrical arrangement of the engine cylinders, the dynamic network of the 3D combustion chamber was composed of eight spray holes. The dynamic 45° sector grid was generated by selecting an oil spray hole. In general, the smaller the grid, the more accurate the prediction of water droplet crushing and evaporation. Cylinder pressure curves for 1 mm, 2 mm, and 4 mm grids, are shown in Fig. 1. When the piston runs to the top dead center, the number of grids in the three grids were 463250, 375500, and 286300, and the calculation times were 15 h, 10 h, and 7 h, respectively. This paper performed mesh encryption near the cylinder wall, piston clearance area, and injector nozzle to guarantee the accuracy of the precision model. As observed in Fig. 1, there was little difference between the cylinder pressure curves of the 2 mm grid and the 1 mm grid. Therefore, the model was simulated on the 2 mm grid to save computational time.

#### 2.2.4 Feasibility test

**Fig. 2:**
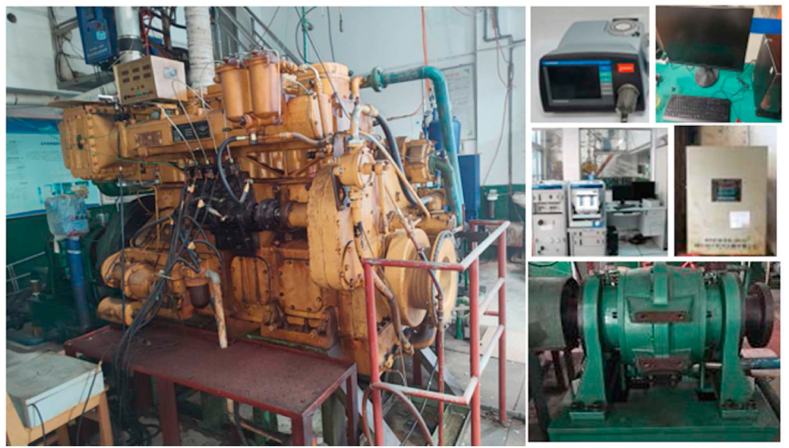
The main equipment of the experimental bench.

As shown in Fig. 2, the modeled object is a four-cylinder, four-stroke medium-speed inline diesel engine. The diesel engine is generally used as a medium-speed power generation diesel engine. Transfer pre-proportioned fuel by volume from the container to the engine fuel tank through an oil pump. The cylinder pressure and NO_x_ emissions were tested at 50% and 100% loads of the engine at a rated speed of 1000 r/min, respectively. Before the start of each test, the diesel fuel system, cooling water system, and other related systems were checked. To ensure steady-state measurements, the measurements were recorded at each operating condition after 25 min. For each test, 15 min were allocated for operation and 10 min for data logging. The experiment was conducted three times and the average value was taken.

**Fig. 3:**
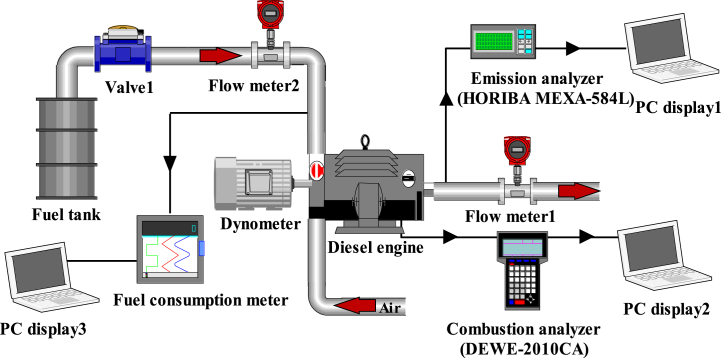
Diagram of the experimental setup.

Before utilizing the simulation model for simulation, it was imperative to verify its accuracy. The experimental setup in this paper is shown in Fig. 3. For example, the ECU was the control unit for the operation of the diesel engine. The dynamometer was utilized to measure the load. Measurements of NO_x_, HC, and CO emissions were taken using an exhaust emission analyzer (HORIBA MEXA-584L), while soot emission was obtained through a smoke opacity meter (AVL Dismoke-4000). The HORIBA MEXA-584L was based on the electrochemical (ELEC) and non-dispersive infrared (NDIR) principles. It was important to preheat the unit for 300 s before measurement and then use the leak check function to check for gas leaks. Finally, followed the steps in the manual to measure the exhaust gas. BSFC was measured by using a fuel consumption meter (FCMM-2). Additionally, the combustion process of the diesel engine was monitored using a combustion analyzer (DEWE-2010CA). The specifications of the used measurement devices are listed in Table 4.

**Table 4:** Specifications of experimental devices.

Device	Content/measured	Precision
Electric dynamometer	NIDY S22-2/05251BV-1	Speed: ± 2r/min; Torque: ±0.8% F.S
Diesel flowmeter	TOCEIL CMFG010	0.1%
Gas flowmeter	TOCEIL 20 N125	±0.8%
Emission analyzer (HORIBA MEXA-584L)	NO, NO_2_ CO HC	±1.0% F.S ±1.0% F.S ±1.0% F.S
Combustion analyzer	DEWE-2010CA	–
AVL Dismoke-4000	Soot emission	±0.001 FSN
Temperature sensor	Thermojunction type	±0.5 °C

#### 2.2.5 Uncertainty analysis

When performing experiments, various factors such as test equipment, environment, conditions, calibration, and readings can impact the results. To ensure accuracy and reliability, an uncertainty analysis is required to understand the extent to which these factors impact the results. Uncertainty for parameters like NO_x_, CO, and BSFC can be calculated using specific equations [54]:

(24) $$V_{R}={\left\{{\left[\left(\partial R/\partial L_{1}\right)\cdot v_{1}\right]}^{2}+{\left[\left(\partial R/\partial L_{2}\right)\cdot v_{2}\right]}^{2}+\cdot \cdot \cdot +{\left[\left(\partial R/\partial L_{n}\right)\cdot v_{n}\right]}^{2}\right\}}^{1/2}$$

(25) $$R=\left\{L_{1},L_{2},L_{3},\cdot \cdot \cdot ,L_{n}\right\}$$

where *V*_*R*_ is the result of uncertainty; *R* is a function of the independent variables *L*_*1*_, *L*_*2*_, …, *L*_*n*_; *v*_*1*_, *v*_*2*_, …, *v*_*n*_ are the uncertainty of the independent variable.

Table 5 lists the measurement ranges and accuracies for a range of measurement objects. Therefore, the overall uncertainty in the experimental process could be obtained by the following equation.

**Table 5:** List of measurements.

Measurements	Measuring range	Accuracy	Uncertainty (%)
NO_x_ emission	0–5000 ppm	±10 ppm	±0.48
CO emission	0–10 %vol	±0.03%	±0.38
HC emission	0–20000 ppm	±10 ppm	±0.12
Soot emission	0–9 FSN	±0.1 FSN	±3.2
Gas flowmeter	0–33.3 kg/min	±0.8%	±0.5
Engine speed	1–2000 rpm	±0.2%	±0.25
BSFC	–	±8 g/kW⋅h	±1.3
Brake power	–	0.05 kW	±0.03
Pressure sensor	0–25 MPa	±10 kPa	±0.5

Total uncertainty of experiment = Square root of [(uncertainty of NO_x_ emissions)^2^ + (uncertainty of HC emission)^2^ + (uncertainty of pressure sensor)^2^ + (uncertainty of brake power)^2^ + (uncertainty of BSFC)^2^ + (uncertainty of CO emissions)^2^ + (uncertainty of soot emissions)^2^] = Square root of [(0.48)^2^+(0.12)^2^ + (0.5)^2^ + (0.03)^2^ + (1.3)^2^ + (0.38)^2^ + (3.2)^2^] = 3.545%

#### 2.2.6 Model validation

**Fig. 4:**
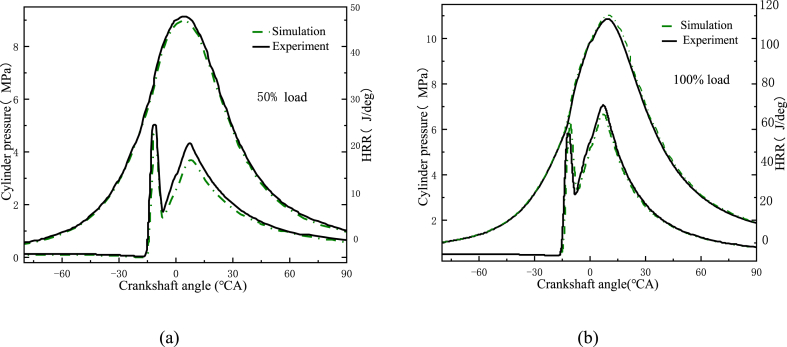
Experimental and simulated curves of cylinder pressure and HRR at different loads. (a) At 50% load; (b) At 100% load.

**Fig. 5:**
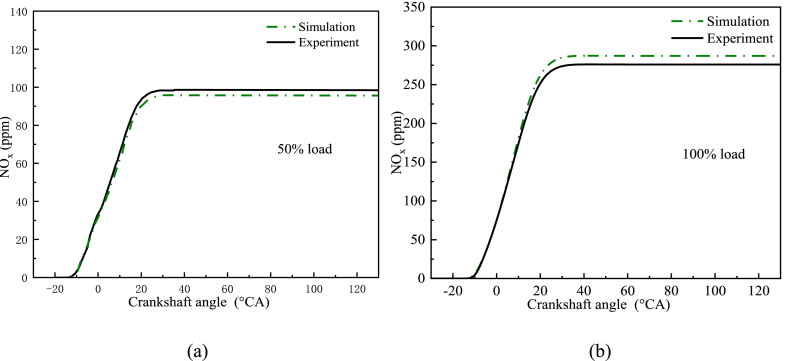
Experimental and simulated curves of NO_x_ emission at different loads. (a) At 50% load; (b) At 100% load.

As shown in Fig. 4 a-b, the experimental and simulation results were compared for cylinder pressure and HRR at 100% and 50% loads. The errors in both the in-cylinder pressure and the ignition process were within 5%. Therefore, the model was accurate. Additionally, Fig. 5 a-b also shows a comparison of simulated and experimental NO_x_ emissions under the 100% and 50% loading conditions. It was observed that the results of the simulation were similar to the experimental ones. As a result, the model could be used to simulate the in-cylinder combustion process.

## 3 Results and discussion

As previously mentioned, biodiesel blends are considered a solution due to diesel shortage and strict environmental standards. Thus, this paper simulates the operation of the diesel engine at 25%, 50%, and 100% loads by the model. This study examined the improvements of engine performance, combustion, and emission characteristics.

### 3.1 Combustion characteristics

#### 3.1.1 Cylinder pressure

**Fig. 6:**
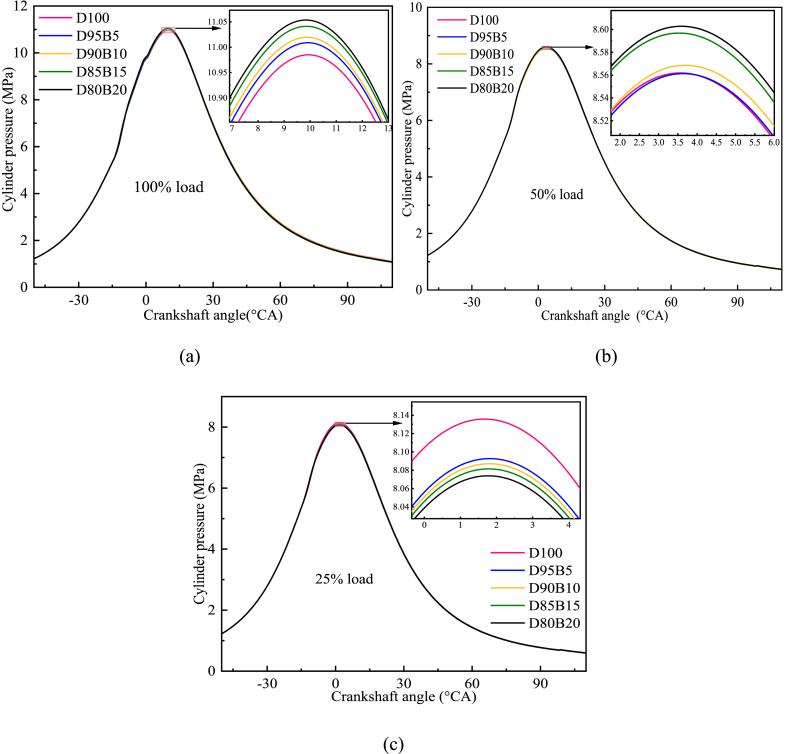
Impact of different biodiesel proportions on cylinder pressure at different loads. (a) At 100% load; (b) At 50% load; (c) At 25% load.

Engine cylinder pressure indicates the efficiency of fuel-air blending [55]. Fig. 6 a-c shows the impact of biodiesel blends with different proportions on cylinder pressure. The results show that the biodiesel blends can effectively improve the maximum cylinder pressure of diesel engines under various load conditions. The highest peak pressure at 100% load is D80B20, followed by D85B15, D90B10, D95B15, and D100. It is because biodiesel contains a higher amount of oxygen compared with diesel. Adding biodiesel increases the blends' oxygen content and promotes fuel combustion in the cylinder [56]. In addition, biodiesel has less calorific value and a higher latent heat of evaporation than pure diesel, leading to an extended ignition delay [57]. Consequently, the cylinder pressure increases with an elevated concentration of biodiesel in biodiesel blends. For example, D80B20 has a 0.6% increase in cylinder pressure at 100% load compared with pure diesel. The same conclusion was reported for biodiesel blends by Duraisamy et al., who noted that biodiesel exhibited the greatest cylinder pressure compared with other fuels tested. This is due to its increased oxygen content and, in particular, enhanced combustion [58]. However, the D100's peak pressure is highest at 25% load. This is because the oxygen content has little impact on cylinder pressure at low loads. But diesel has a higher calorific value compared with biodiesel [59]. As the biodiesel blend proportion increases, the peak cylinder pressure decreases. It may be impacted by the lower heating value of biodiesel. Elsharkawy et al. [60] and Zhang et al. [61] came to similar conclusions. By controlling the amount of biodiesel added and reasonably increasing the cylinder pressure, the combustion efficiency of the fuel blend can be further improved and the pollutant emission level decreased.

#### 3.1.2 Cylinder temperature

**Fig. 7:**
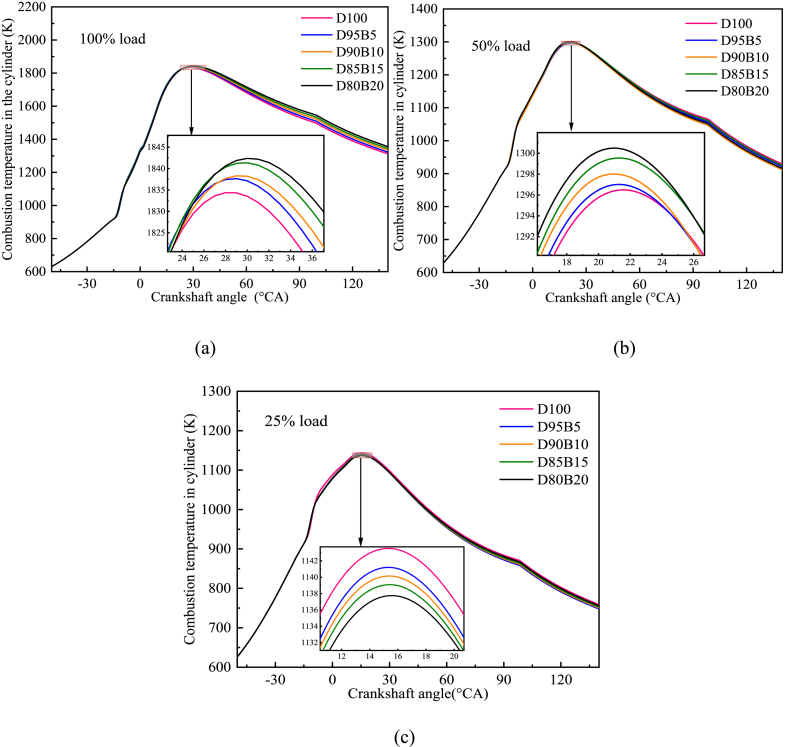
Impact of different biodiesel proportions on cylinder temperature at different loads. (a) At 100% load; (b) At 50% load; (c) At 25% load.

Fig. 7 a-c shows the impact of different proportions of biodiesel blends on cylinder temperature. It has been observed that during the high-load operation of diesel engines, the peak combustion temperature in the cylinder tends to increase with the increase of biodiesel blend proportion. Specifically, D80B20 has the highest peak temperature at 100% load, which is followed by D85B15, D90B10, D95B5, and D100. The key factor is that the inclusion of biodiesel enhances the total oxygen level of the blends. The increase in oxygen content provides a more effective combustion process in the cylinder [62]. However, the level of oxygen present in the blends has a minimal impact on the combustion process when the diesel engine is operated at a low load condition. This is possibly caused by the reduction in heat release per unit volume of fuel blends when biodiesel is added. This results in a lower temperature in the cylinder. Zhang et al. [63] obtained similar results. By measuring the cylinder temperature variation under different blending proportions, the combustion process of biodiesel blends can be explored to provide a theoretical reference for optimizing fuel proportions.

**Fig. 8:**
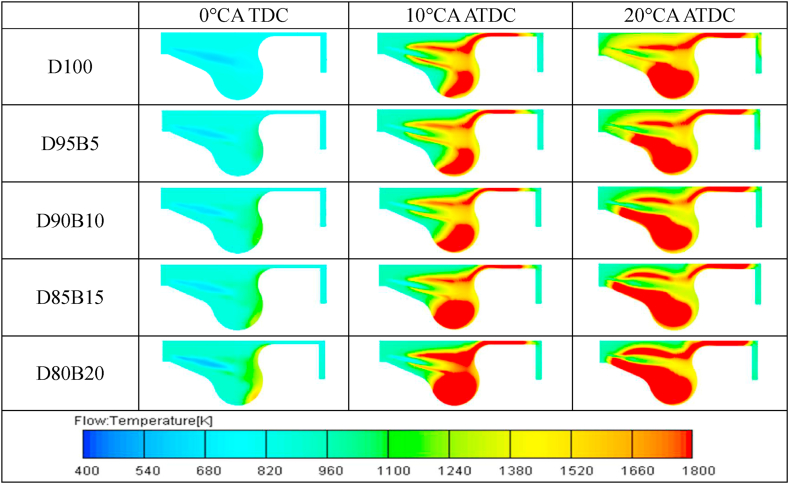
Temperature field distribution of diesel-biodiesel blends at 50% load.

Fig. 8 shows the distribution at 50% load of the temperature of the combustion field in the cylinder for the five fuels in a diesel engine. The results show that the local high-temperature region in the diesel engine cylinder gradually increases with the increase of the proportion of biodiesel in the blends. The reason for this is that the high oxygen content of biodiesel promotes complete combustion of the fuel [64]. Therefore, the cylinder temperature is increased. In addition, the spray penetration of biodiesel is decreased due to the high viscosity of biodiesel [61]. As a result, biodiesel will produce more local high temperature region.

### 3.2 Economic characteristics

#### 3.2.1 Brake power

**Fig. 9:**
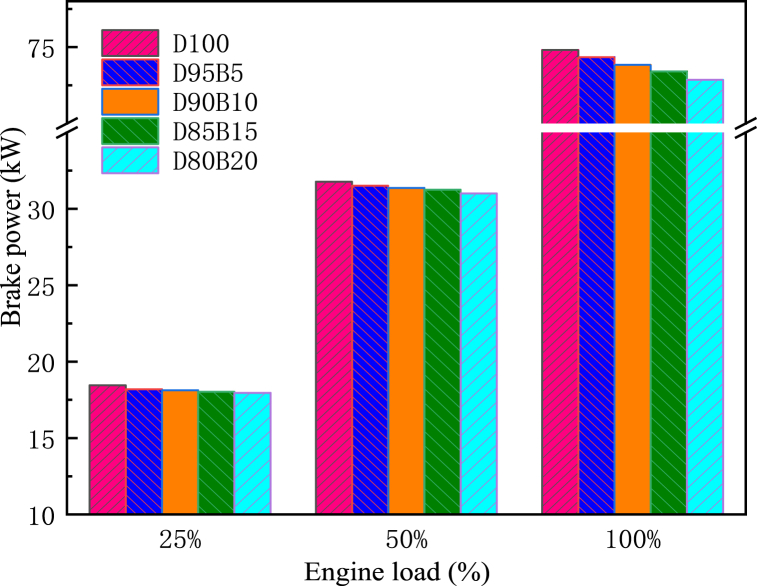
Impact of different biodiesel proportions on BP at different loads.

The BP of an engine is the rate at which it performs its work. The calorific value and viscosity of the fuel tend to impact the engine's BP [65]. Fig. 9 shows the impact of biodiesel blends with different proportions on BP. The results showed that BP increases with increasing diesel engine load. Specifically, D100 has the largest BP at all loads, followed by D95B5, D90B10, D85B15, and D80B20. The BP of D95B5, D90B10, D85B15, and D80B20 blends are 0.64%, 1.31%, 1.88%, and 2.62% lower than D100 at 100% load, respectively. For biodiesel blends, Emaish et al. reported that fuel blend proportion had a significant impact on BP, with pure diesel having the highest BP and 100% biodiesel having the lowest BP compared with other blend proportions [66]. The reason is that the total calorific value of the blends decreases as the proportion of biodiesel increases [67]. This leads to a decrease in temperatures and pressures within the cylinders, resulting in a reduction of the BP. Gongora et al. [68] had similar conclusions. By comparing BP at different biodiesel blending proportions, it provides a basis for selecting the best biodiesel proportion. It is known from this study that the lower proportion of biodiesel has a less negative impact on BP.

#### 3.2.2 Brake specific fuel consumption

**Fig. 10:**
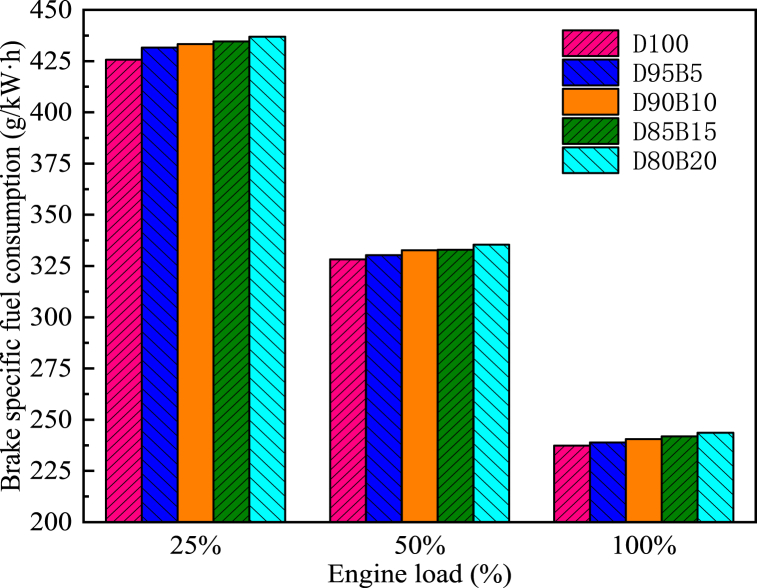
Impact of different biodiesel proportions on BSFC at different loads.

One of the most significant factors in determining fuel quality is BSFC, which assesses fuel energy and energy efficiency [69]. Diesel engines' BSFC decreases as the load increases, as shown in Fig. 10. This is due to the fact that the engine wall surface is hot enough to atomize better, burn and generate maximum heat [70]. Therefore, the BSFC is decreased. However, the BSFC of diesel engines increases with an increase in biodiesel content in blends. For instance, compared with D100, the BSFCs for D95B5, D90B10, D85B15, and D80B20 were increased by 0.61%, 1.34%, 1.42%, and 2.17%, respectively at 50% load. This phenomenon is common in oxygenated fuels such as biodiesel [71]. The reason is that the biodiesel has a lower heating value. Therefore, the diesel engine requires a more substantial quantity of fuel under the same conditions to fulfill its operational prerequisites [72]. Cihan et al. [73] reported that the BSFC value increased with increasing biodiesel usage because of the lower calorific value and lower cetane number of biodiesel fuel. Alahmer et al. [74] arrived at comparable findings. It can be concluded that the higher the proportion of biodiesel the greater the negative impact on BSFC.

#### 3.2.3 Brake thermal efficiency

**Fig. 11:**
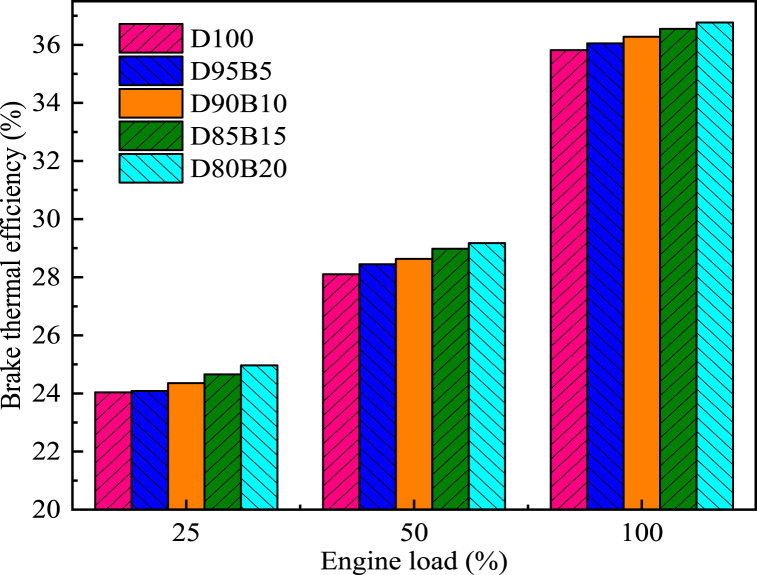
Impact of different biodiesel proportions on BTE at different loads.

BTE is a significant parameter and used to measure the energy conversion efficiency of the engine. Thus, it plays a key role in characterizing the economy of a given engine [75]. Fig. 11 shows the impact of biodiesel blends with different proportions on BTE. The results indicate that BTE increases with an increase in the load of the diesel engine. It is due to the improved combustion and decreased friction losses at higher loads [76]. The BTE of biodiesel blends is higher compared with D100, and there is a trend that the higher the proportion of biodiesel in the blend, the higher the BTE. For instance, at 50% load, D80B20 has the highest BTE, followed by D85B15, D90B10, D95B5, and D100. Specifically, the BTE of the biodiesel blends D95B5, D90B10, D85B15 and D80B20 increased by 1.24%, 1.89%, 3.13% and 3.82%, respectively, compared with D100. This is attributed to the high oxygen content of biodiesel that allows the fuel to combust completely. This means that more energy is produced when the same amount of fuel is combusted. In addition, when the engine load increases, the cylinder temperature increases to improve the spray characteristics and improve the cylinder combustion [77]. Madane et al. [78] and Sajjad et al. [79] arrived at comparable findings. It can be concluded that the combustion process of biodiesel blends in diesel engines can be improved by adjusting the blending proportion, thus increasing the BTE.

### 3.3 Emission characteristics

#### 3.3.1 NO_x_ emissions

**Fig. 12:**
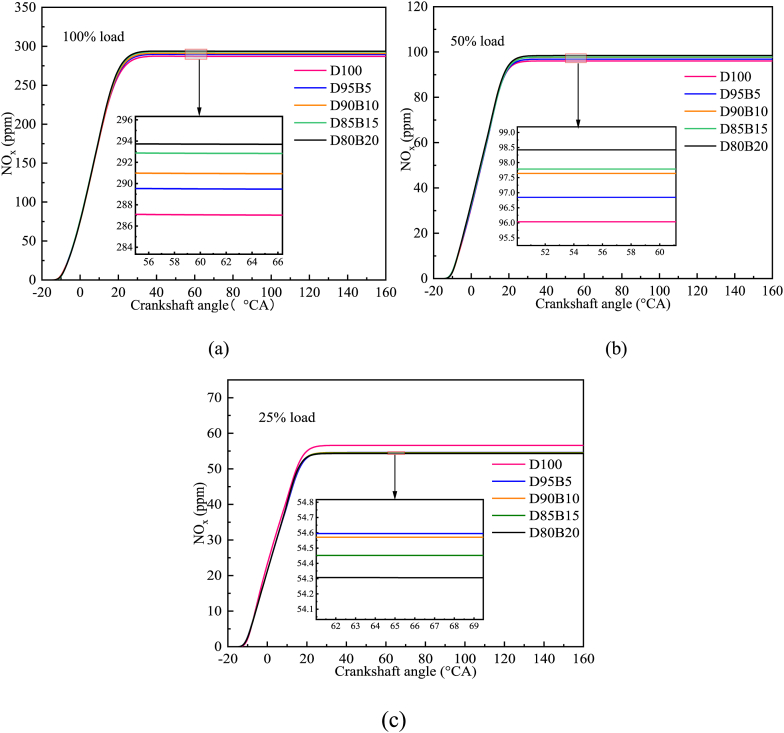
Impact of different biodiesel proportions on NO_x_ emissions at different loads. (a) At 100% load; (b) At 50% load; (c) At 25% load.

NO_x_ emissions from diesel engines are strongly impacted by cylinder temperature, oxygen content, and combustion time. Fig. 12 a-c shows the impact of different biodiesel blend proportions on NO_x_ emissions. As engine load increases, NO_x_ emissions generally increase. This correlation can be attributed to the lower cylinder temperature at low loads resulting in less NO_x_ production. As the load increases, the air-fuel ratio decreases, resulting in higher engine cylinder temperatures and longer fuel burn times [80]. It causes an increase in NO_x_ emissions. This situation is consistent with the results reported by Nabi et al. [81]. Furthermore, NO_x_ emissions at 50% and 100% loads are found to increase with higher biodiesel proportions in fuel blends. For instance, D80B20 showed the most NO_x_ emissions at 100% load, followed by D85B15, D90B10, D95B5, and D100. This trend is similar to the study by Reksowardojo et al. [31], who reported that as the proportion of biodiesel increases, NO_x_ emissions increase, and pure biodiesel fuel has the highest NO_x_ content. This phenomenon is attributed to the higher oxygen content in the blend from biodiesel, leading to the increase of NO_x_ production. At 25% load, NO_x_ emissions decrease as the proportion of biodiesel in the biodiesel blend increases. The low calorific value of biodiesel results in lower cylinder temperatures, which results in lower NO_x_ emissions. NO_x_ emissions at this point are inversely proportional to the proportion of biodiesel in the blends. Moreover, the evaporation heat of biodiesel additionally decreases cylinder temperature, further decreasing NO_x_ emissions. Huang et al. [82] reached similar conclusions. Based on this study, it can provide a reference for the development of appropriate fuel blends to suppress NO_x_ emissions.

#### 3.3.2 CO emissions

**Fig. 13:**
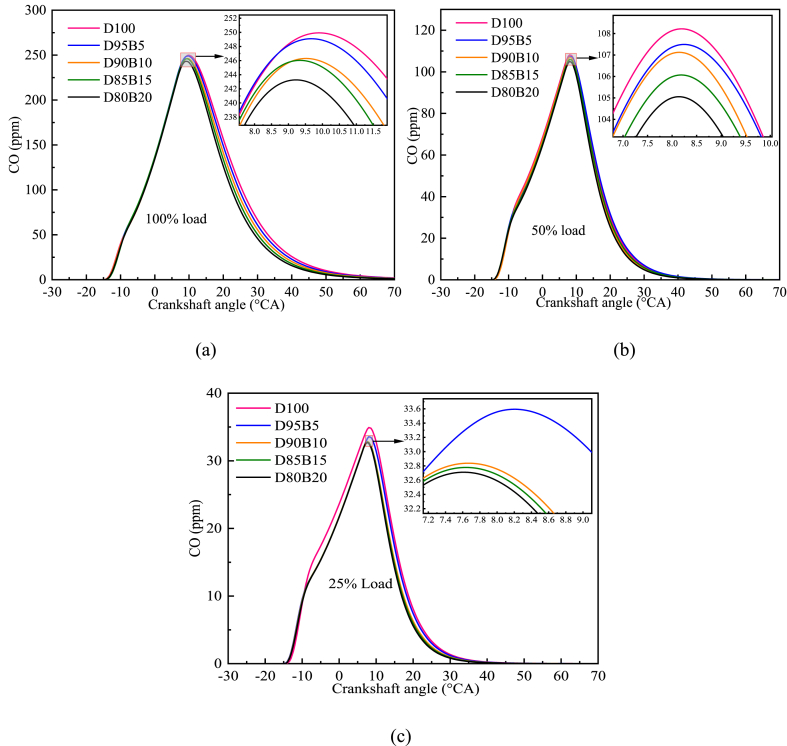
Impact of different biodiesel proportions on CO emissions at different loads. (a) At 100% load; (b) At 50% load; (c) At 25% load.

The main reason for producing CO is incomplete combustion [83]. The effective oxidation of CO is an important measure to decrease its emissions [84]. Fig. 13 a-c shows the impact of biodiesel blends with different proportions on CO emissions. The results show that as the engine load increases, the amount of CO production also increases. This is because the increase in load leads to an increase in cylinder temperature, which facilitates complete combustion [85]. For all loads, CO emissions decrease as the proportion of biodiesel in the blends increase. For instance, D100 has the highest CO emissions at 100% load, followed by D95B5, D90B10, D85B15, and D80B20. Notably, the CO emissions of D80B20 blend are 2.71% lower compared with D100. It is due to the biodiesel's high oxygen content that promotes complete combustion [86]. In addition, the higher oxygen content of biodiesel enhances the oxidation of CO to CO_2_, which is beneficial for reducing the production of CO [87]. Madhu et al. [88] and Mahmood et al. [89] arrived at comparable findings. According to this study, it is known that increasing the proportion of biodiesel can reduce CO emissions and provide a reference basis for controlling CO emissions.

#### 3.3.3 Soot emissions

**Fig. 14:**
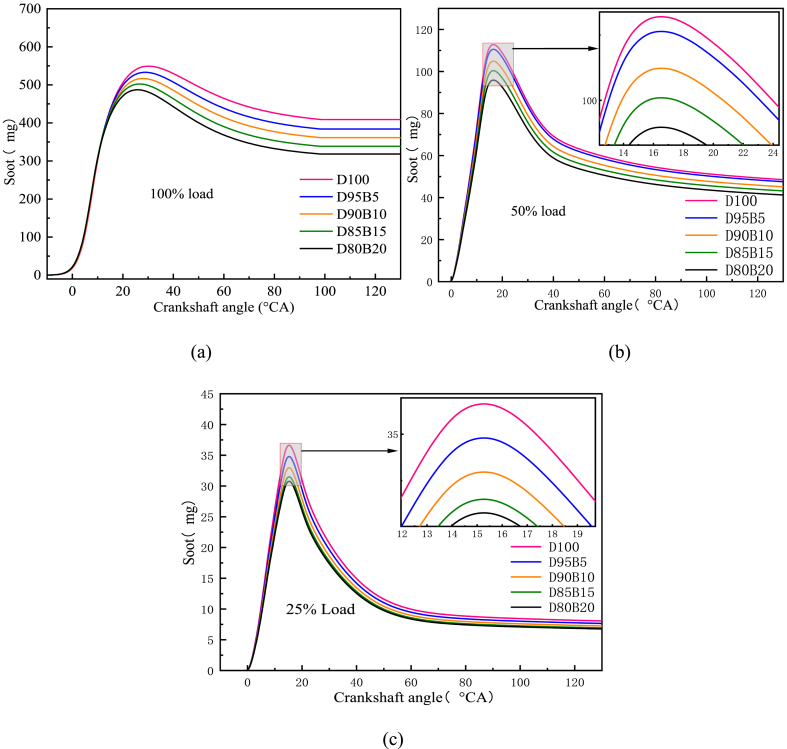
Impact of different biodiesel proportions on soot emissions at different loads. (a) At 100% load; (b) At 50% load; (c) At 25% load.

Fig. 14 a-c shows the impact of biodiesel blends with different proportions on soot emissions. It has been observed that the emissions of soot are directly proportional to the load on diesel engines. This is mainly due to the increase in the amount of fuel sprayed per cycle as the engine load increases. As a result, the excess air factor decreases and the concentration of the fuel blend increases, leading to more fuel enriched areas and incomplete combustion [90]. This increases the level of soot emission in the exhaust of diesel engines. When the diesel engine load decreases, the in-cylinder combustion temperature also decreases. At the same time, the air-fuel proportion becomes larger. The result is a longer oxidation time for in-cylinder fuel combustion to generate soot, which lowers soot emissions. Additionally, soot emissions decreased with increasing biodiesel fuel blend at all loads. For instance, at 50% load, the diesel engine had the highest soot emissions when combusting D100. Compared with D100, the soot emissions for a diesel engine were decreased by 1.20%, 2.64%, 3.88%, and 4.65% for 95B5, D90B10, D85B15, and D80B20 blends, respectively. The main reason for this is that the oxygen atoms contained in biodiesel act as a combustion aid during the combustion process, promoting the complete combustion of the blends and accelerating the oxidation of soot. Other possible reasons for the decrease in soot emissions are the decrease in carbon/hydrogen proportion and the decrease in aromatics and sulfur content with the addition of biodiesel [91]. Adrian et al. [92] and Zhang et al. [93] reached similar conclusions. It can be concluded that the combustion process of biodiesel blends in diesel engines can be improved by adjusting the blending proportion, thus decreasing soot emissions.

## 4 Conclusions

Biodiesel is potential renewable alternative fuel for automobiles [94]. In the paper, a 3D simulation model was developed by using CONVERGE simulation software and the improvements for a four-cylinder fueled biodiesel blends diesel engine were investigated in term of the cylinder pressure, cylinder temperature, BP, BTE, BSFC, NO_x_, CO, and soot emissions. The main research contents and conclusions are summarized as follows.

(1) The engine's economic characteristics can be significantly impacted by the use of biodiesel blends. Compared with D100, biodiesel blends can increase the BTEs of diesel engines. It is due to the improvement combustion caused by the oxygen content in the biodiesel. However, the increase of the proportion of biodiesel decreases the total calorific value of the biodiesel blend. Therefore, the addition of biodiesel decreases BP and increases BSFC in diesel engines compared with pure diesel.
(2) The proportion of biodiesel in biodiesel blends plays a very important role. When the engine operates at 50% and 100% load, higher biodiesel concentrations in the biodiesel blend result in the increases of in-cylinder temperature and pressure peaks. This is because the high oxygen content of biodiesel enhances combustion. However, at 25% load, the oxygen content of the biodiesel blend has relatively little impact on combustion. Since the calorific value of biodiesel is lower than that of diesel, the addition of biodiesel leads to a lower heat release per unit volume of biodiesel blend. This in turn leads to lower in-cylinder combustion temperatures and pressure peaks as the biodiesel proportion increases.
(3) The biodiesel in biodiesel blends can significantly impact engine emissions. As the proportion of biodiesel in the biodiesel blend increases, soot, and CO emissions decrease at all loads. Compared with D100, the soot emissions for a diesel engine were decreased by 1.20%, 2.64%, 3.88%, and 4.65% for D95B5, D90B10, D85B15, and D80B20 blends, respectively. This is caused by the higher oxygen content in biodiesel, which promotes more complete fuel combustion. However, NO_x_ emissions can slightly increase due to the higher oxygen content and cylinder temperature that enhance NO_x_ production.

In future work, other additives and methods can be used to further improve the performance of biodiesel. Biodiesel has great potential and promise for commercial application from the perspective of sustainability and environmental protection. In summary, the application of biodiesel as a partial substitute for diesel in diesel engines can significantly decrease the emission of exhaust pollutants. It has important research significance for alleviating the energy crisis and decreasing environmental pollution. In addition, the results of this study provide valuable information for further optimization of biodiesel blends.

## Author contribution statement

Yanhui Chen: Conceived and designed the experiments; Performed the experiments; Contributed reagents, materials, analysis tools or data.

Jian Zhang: Performed the experiments; Wrote the paper.

Zhiqing Zhang: Conceived and designed the experiments; Contributed reagents, materials, analysis tools or data; Wrote the paper.

Weihuang Zhong: Ziheng Zhao: Jingyi Hu: Analyzed and interpreted the data.

## Data availability statement

Data will be made available on request.

## Funding statement

The work is supported by the Guangxi key research and development Foundation under the research grant 2023AB01092.

## Declaration of competing interest

The authors declare that they have no known competing financial interests or personal relationships that could have appeared to influence the work reported in this paper.
